# Domain knowledge integration into deep learning for typhoon intensity classification

**DOI:** 10.1038/s41598-021-92286-w

**Published:** 2021-06-21

**Authors:** Maiki Higa, Shinya Tanahara, Yoshitaka Adachi, Natsumi Ishiki, Shin Nakama, Hiroyuki Yamada, Kosuke Ito, Asanobu Kitamoto, Ryota Miyata

**Affiliations:** 1 Graduate School of Engineer and Science, University of the Ryukyus, Nishihara-cho, Nakagami-gun Okinawa, Japan; 2 Faculty of Engineering, University of the Ryukyus, Nishihara-cho, Nakagami-gun Okinawa, Japan; 3 Faculty of Science, University of Ryukyus, Nishihara-cho, Nakagami-gun Okinawa, Japan; 4 Digital Content and Media Sciences Research Division, National Institute of Informatics, Chiyoda-ku, Tokyo, Japan

**Keywords:** Climate sciences, Computational science, Computer science

## Abstract

In this report, we propose a deep learning technique for high-accuracy estimation of the intensity class of a typhoon from a single satellite image, by incorporating meteorological domain knowledge. By using the Visual Geometric Group’s model, VGG-16, with images preprocessed with fisheye distortion, which enhances a typhoon’s eye, eyewall, and cloud distribution, we achieved much higher classification accuracy than that of a previous study, even with sequential-split validation. Through comparison of t-distributed stochastic neighbor embedding (t-SNE) plots for the feature maps of VGG with the original satellite images, we also verified that the fisheye preprocessing facilitated cluster formation, suggesting that our model could successfully extract image features related to the typhoon intensity class. Moreover, gradient-weighted class activation mapping (Grad-CAM) was applied to highlight the eye and the cloud distributions surrounding the eye, which are important regions for intensity classification; the results suggest that our model qualitatively gained a viewpoint similar to that of domain experts. A series of analyses revealed that the data-driven approach using only deep learning has limitations, and the integration of domain knowledge could bring new breakthroughs.

## Introduction

Because typhoons, or tropical cyclones (TCs), are among the world’s most destructive natural disasters, accurate classification of their intensity has been particularly important in the field of weather forecasting^1–4^. As typhoons spend most of their lives at sea, away from land, stable observations are often available only from satellites. Therefore, the Dvorak technique and similar methods^5–9^ are widely used to quantitatively estimate the intensity class of a typhoon solely according to visible and infrared satellite images. The future evolution of satellite-based typhoon intensity classification methods is of vital interest to meteorologists and coastal communities, and continued improvement should be a top research priority in the atmospheric sciences^10^. The difficulty of the Dvorak technique lies in extracting the key features of cloud distributions that are related to the typhoon intensity from a satellite image. The current methodology relies strongly on the experience and intuition of experts. Moreover, changes in operational procedures and observational capabilities over time and between meteorological agencies have introduced heterogeneities in the estimation of typhoon intensity classes^11–16^.

In order to reduce the classification ambiguity, there have been several attempts to automate the Dvorak technique by using deep learning models that have achieved breakthroughs in image recognition^17–28^ (for review, see^29^). In one representative work, Pradhan et al.^18^ designed a convolutional neural network (CNN) architecture for estimating the hurricane intensity category. Following the common practice in machine learning, they performed k-fold validation, which randomly split the original dataset into a training set and a test set, resulting in a high classification accuracy of 82%. In contrast, to prevent data leakage, time-series data such as typhoon images should normally be split sequentially so that the timestamps of the training data are earlier than those of the test data^30^. However, random split validation with time-series data normally yields better-than-real-world performance, because it enables the model to learn future trends that are supposed to be unavailable in the training process. Unfortunately, in our preliminary experiment^31^ using a typhoon intensity classification model with an architecture similar to that of Pradhan et al.^18^, we found that the accuracy dropped to 55.7% when a sequential split was adopted instead of a random split (for details, see Supplementary Fig. S1).

After Pradhan et al. (2018), many studies proposed methodologies to use a CNN or a more sophisticated deep learning model to analyze satellite imagery and estimate TC intensity^19–28^. Recent trend is to input a combination of satellite images and other information into CNNs. Chen et al.^21^ proposed a CNN architecture to estimate TC intensity from infrared (IR1) brightness temperatures and passive microwave (PMW) rain rates. They concluded that their best model was competitive with existing methods such as the advanced Dvorak technique (ADT^32^) and Satellite Consensus (SATCON^33^). Lee et al.^27^ used a two-dimensional CNN (2D-CNN) and a three-dimensional CNN (3D-CNN) to analyze the relationship between multispectral geostationary satellite images and TC intensity. Their optimal model exhibited better performance (35%) than the existing model using the CNN-based approach with a single channel image. Their methods are very powerful and sophisticated, but they have not been able to answer the question of how much information about typhoon intensity can be maximally extracted from a single satellite image. Moreover, to obtain a sufficient number of training samples, most studies that directly use a CNN to estimate typhoon intensity perform data expansion by randomly rotating or adding Gaussian noise to the original satellite imagery. However, the resulting expanded dataset contains images that are unnatural as typhoons, and the CNN consequently acquires a completely different view from that of humans, making it difficult to interpret the CNN’s criteria for estimation.

In that case, how can we improve the performance of a classification model based on a single satellite image? Our first idea is to use a “deeper” CNN^34–37^. The architecture of Pradhan et al.^18^ is based on LeNet^38^, which is an extremely popular and simple CNN; however, CNNs with smaller convolution filters (or receptive fields), which consequently increase the network depth, may be better suited to extract more detailed cloud patterns of a typhoon from a satellite image. Our second idea is to use domain knowledge for image preprocessing. Through discussions with meteorological experts, we learned that when looking at a satellite image of a typhoon, they pay attention to whether the characteristics of a violent typhoon (the highest intensity level) appear in the image, such as clear visibility of the typhoon’s eye and a concentric cloud distribution around the eye. Therefore, we hypothesize that by applying fisheye distortion^39^ to a satellite image to enhance the typhoon’s eye, eyewall, and cloud distribution around the center, we might enable a CNN to “see” the typhoon image similarly to how domain experts see it, resulting in intensity class estimation with a higher accuracy.

In this study, we propose an alternative automated Dvorak technique that uses a deep learning model by the Visual Geometrical Group (VGG), called VGG-16^40^, and image preprocessing based on meteorological domain knowledge. By using VGG-16 with fisheye-preprocessed satellite images, we achieve much higher classification accuracy, even with sequential-split validation, than in the previous study^18^. Then, we evaluate the classification potential of the feature maps in our model by using t-distributed stochastic neighbor embedding (t-SNE)^41^. Finally, by using gradient-weighted class activation mapping (Grad-CAM)^42^, we visualize how our model views typhoon images and confirm its qualitative match to the estimation of meteorological experts. Because we also aim to interpret the rough image features of dangerous typhoons (e.g., very strong and violent typhoons) by using Grad-CAM, we treat the problem of intensity estimation primarily as a classification task. Even today, several broad categories of typhoon intensity classification are used to provide information to the general public, and we hope that our analysis will contribute to disaster prevention.

**Figure 1:**
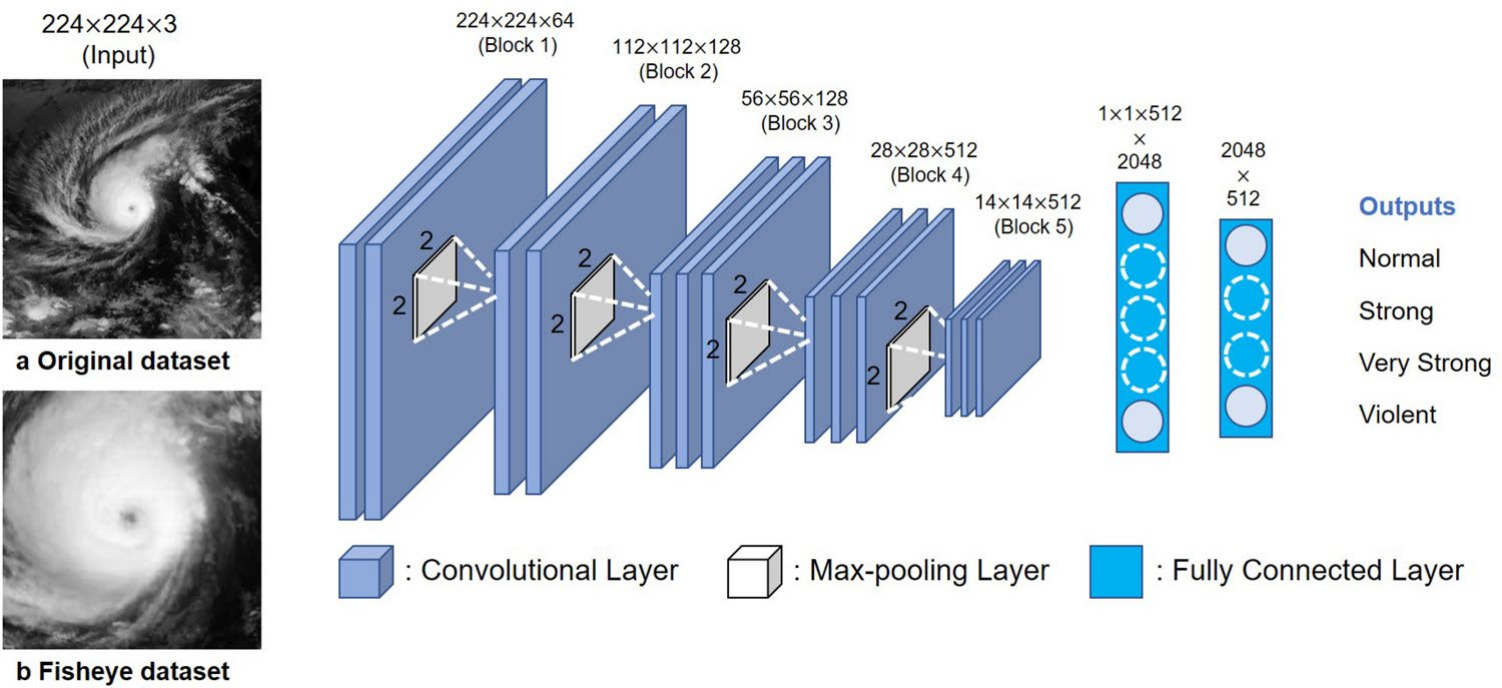
VGG-16 architecture used in this paper for typhoon intensity classification from satellite images. Two kinds of satellite images of typhoons were given to the model and compared in terms of the resulting classification accuracy: (**a**) the original images and (**b**) fisheye-preprocessed images.

**Table 1:** Summary of the typhoon intensity definitions and the sizes of the training and testing datasets.

Category	Symbol	Wind speed (knots)	Training	Testing
Normal	N	34–63	2796	659
Strong	S	64–84	1018	303
Very strong	VS	85–104	633	227
Violent	V	105	134	46

## Results

At the beginning, it should be mentioned that this study only focuses on the western North Pacific TCs (i.e., typhoons). We estimated the typhoon intensity classes by using two models: (a) VGG-16 with the original satellite images and (b) VGG-16 with the fisheye-preprocessed images. The CNN architecture is shown in Fig. 1. Table 1 summarizes the Japan Meteorological Agency (JMA) definitions of the four typhoon intensity classes^45^, which are the classification target for the CNN output. Following these definitions, we converted the best-track maximum sustained wind (MSW, 10-min mean) associated with a typhoon, which was obtained from the Regional Specialized Meteorological Center (RSMC) Tokyo^46^, into the intensity. Subsequently, the typhoon images were labeled with the corresponding intensity classes. For each classification model, we used sequential-split validation: the data of typhoons that occurred from 2005 to 2014 was used for training, while that from 2015 to 2016 was used for testing. The detailed hyperparameters of the proposed models are shown in Supplementary Table S1.

Table 2a shows a confusion matrix of the typhoon intensity classification when VGG-16 was fed the original dataset. The gray cells indicate the numbers of typhoon images that were correctly classified with the model. As seen in this table, the classification accuracy improved by 13.9% (i.e., to an accuracy of 68.9%) as compared to our previous study^31^. However, there remain some serious underestimations of typhoon intensity: VGG-16 with the original images misclassified two violent typhoons as normal ones. On the other hand, Table 2b shows a confusion matrix of the typhoon intensity classification for the fisheye-preprocessed image dataset. Note that the results listed in Table 2b were obtained using the same VGG-16 model architecture used for the results listed in Table 2a. By comparing the tables, we can see that the fisheye-preprocessed images enabled VGG-16 to classify the typhoon intensity more precisely than it could with the original images, resulting in a classification accuracy of 76.8%.

We also performed regression analysis to estimate the speed of sustained winds by using the following equation:

1 $$\begin{aligned} y = \beta _0 + \beta _1 x_1 + \beta _2 x_2 + \beta _3 x_3 + \beta _4 x_4. \end{aligned}$$

**Figure 2:**
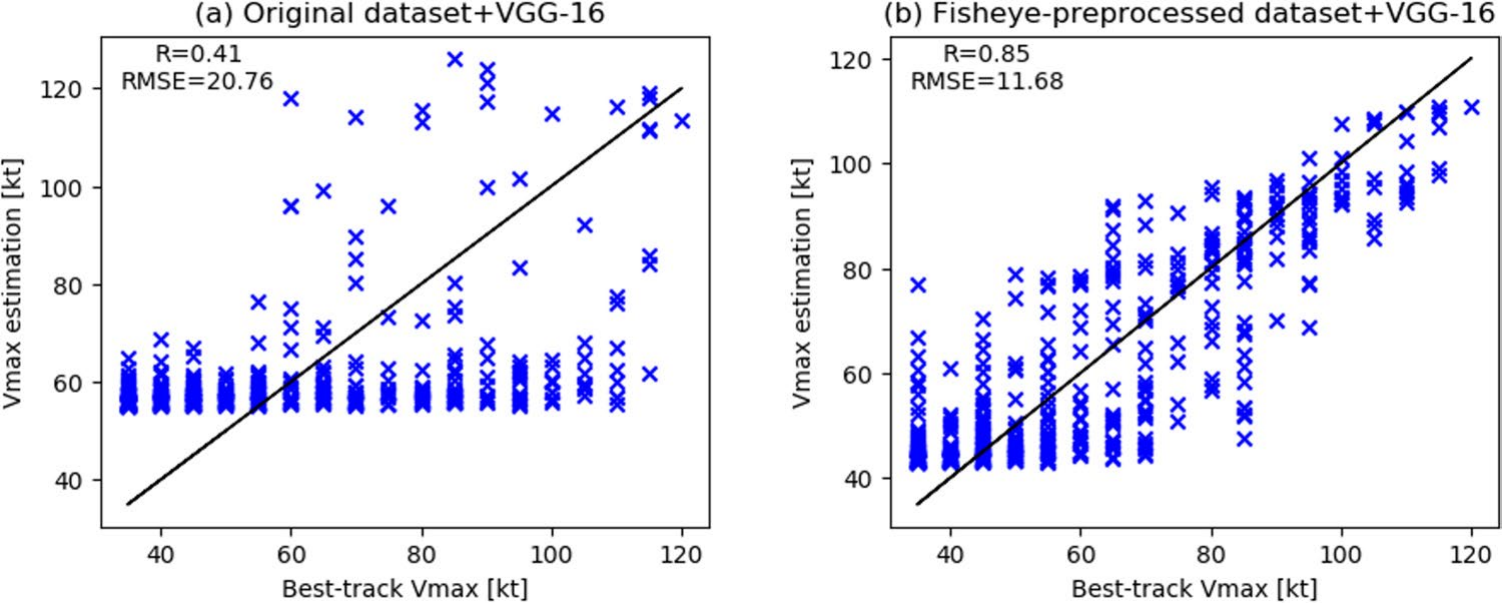
Scatter plots of linear regressions to estimate the speed of sustained winds (10-min mean) from the predicted probabilities of each typhoon intensity class output by the proposed models.

Here, *y* is the best-track MSW (10-min mean) associated with a typhoon, the $\beta _{i}~(i=0,~1,~{\cdots },~4)$ are the regression coefficients, and the $x_{j}~(j=1,2,3,4)$ represent the prediction probabilities of each intensity class output by the proposed models shown in Fig. 1 (1: normal; 2: strong; 3: very strong; 4: violent). We provided two types of explanatory variables $x_{j}$: (a) the prediction probabilities output by VGG-16 after it was well trained with the original images of typhoons from 2005–2014, and (b) the prediction probabilities output after training with the fisheye-preprocessed images. For each linear regression model, the JMA best-track data for typhoons in 2015 was used for training, while that in 2016 was used for testing. We then compared the root-mean-square error (RMSE) and the correlation coefficient *R* between the two regression models. Figure 2 shows the results of the regression analysis. Whereas the intensity RMSE of the regression model based on the original images was 20.76 kt, with $R=0.41$, that of the model based on the fisheye-preprocessed images was 11.68 kt, with $R=0.85$. Furthermore, comparing Fig. 2a and b, we can see that the regression model based on the fisheye images had smaller errors than the model based on the original images, especially for typhoons with wind speeds of 90 kt or higher.

**Table 2:** Confusion matrices and classification results for VGG-16 with the (**a**) original and (**b**) fisheye-preprocessed datasets.

Actual category	Prediction						
	N	S	VS	V	Precision	Recall	F1-score
**(a) Original dataset + VGG-16 (accuracy:68.9%)**							
Normal	602	54	3	0	0.76	0.91	0.83
Strong	141	131	31	0	0.50	0.43	0.46
Very strong	44	75	105	3	0.63	0.46	0.53
Violent	2	4	27	13	0.81	0.28	0.42
**(b) Fisheye-preprocessed dataset+ VGG-16 (accuracy:76.8%)**							
Normal	598	61	0	0	0.84	0.91	0.87
Strong	95	192	16	0	0.6	0.63	0.62
Very strong	16	64	126	21	0.83	0.56	0.66
Violent	0	3	10	33	0.61	0.72	0.66

Furthermore, we developed a CNN regression to estimate the best-track MSW (10-min mean) value of a typhoon directly from a single satellite image without using any classifier. The CNN architecture is shown in Supplementary Fig. S2(a), which is the same VGG-16 as Fig. 1 except for the output layer. The same two types of satellite images (i.e., (a) the original and (b) fisheye-preprocessed datasets) as in the proposed classification model were respectively input to the regression model, and the same sequential split validation was adopted (i.e., data from 2005 to 2014 was for training and those from 2015 to 2016 was for testing). Figure S2(b) shows the results of the CNN regression. Similar to Fig. 2, VGG-16 with the fisheye-preprocessed images had better estimation accuracy than that with the original images: Whereas the RMSE of VGG-16 regression with the original images was 13.97 kt, with $R=0.79$, that with the fisheye-preprocessed images was 11.5 kt, with $R=0.85$. We then classified the wind speed estimated by the VGG regression into four categories according to the typhoon intensity definitions shown in Table 1. The results are shown in Supplementary Fig. S3(c), which is very similar to those in Table 2: The VGG-16 with the fisheye-preprocessed images had higher accuracy and recall for violent typhoons than that with the original images.

**Figure 3:**
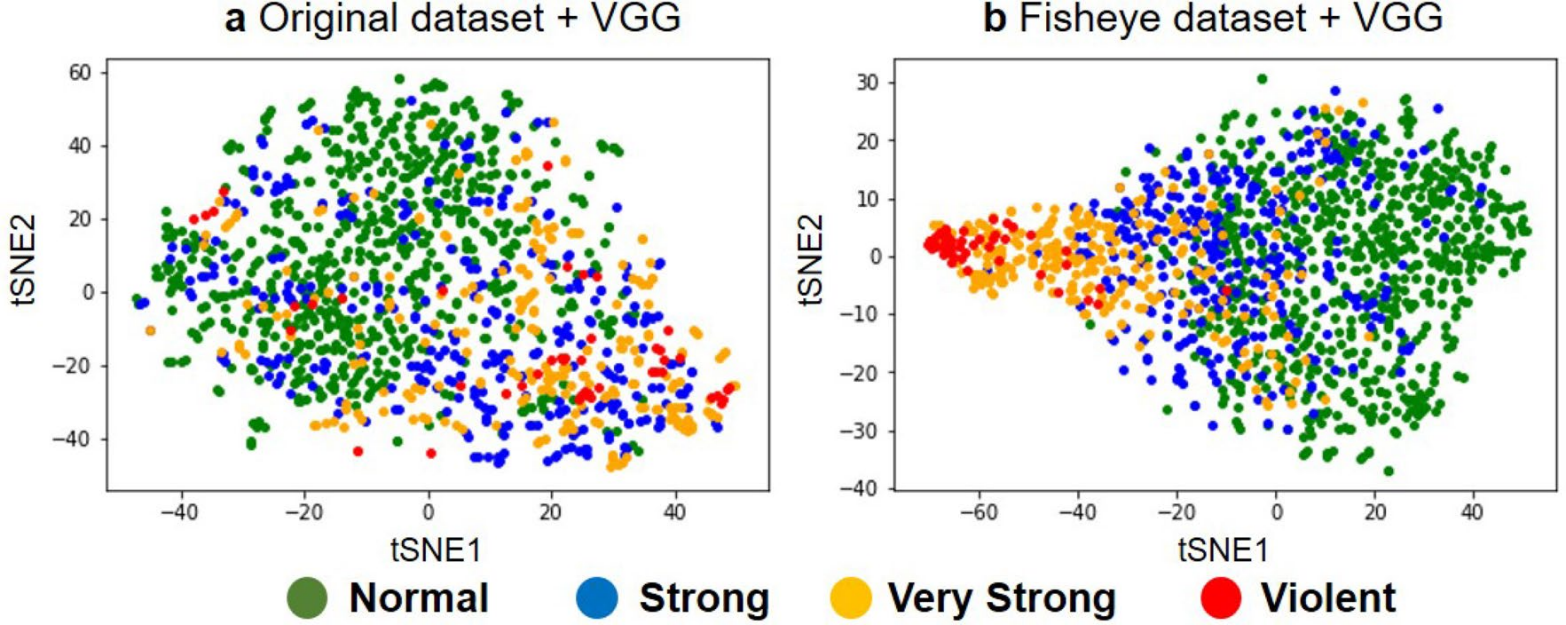
t-SNE visualization of the feature maps extracted from the last convolutional layer in VGG-16 for the **(a)** original and **(b)** fisheye-preprocessed test datasets.

## Discussion

### t-SNE visualization

To understand why VGG-16 achieved a higher accuracy of more than 76.8% with the fisheye-preprocessed images, we evaluated the classification potential of the feature maps by using t-SNE. Specifically, we visualized the distributions of the outputs from the last convolutional layer (i.e., the inputs to the first fully connected layer) in each model when given the test data. As shown in Fig. 3a, it was difficult for VGG-16 with the original images to completely distinguish the intensity classes, as the data points were not separated well by intensity class in the two-dimensional space. In contrast, Fig. 3b shows a significant improvement by using the fisheye-preprocessed images: the data points were clustered well in their intensity classes. This result suggests that the fisheye preprocessing, which enhances the typhoon eye and the cloud distribution near the center of an image, enabled VGG-16 to distinguish the typhoon intensity classes more sharply.

**Figure 4:**
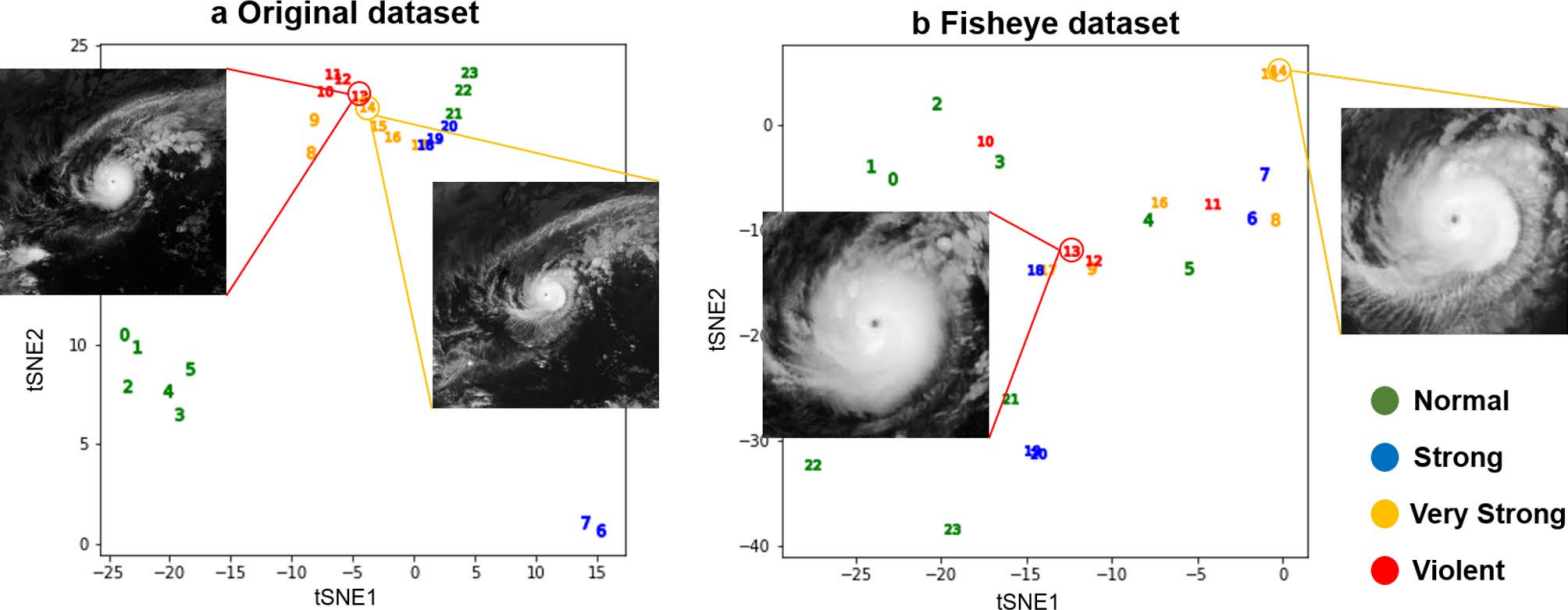
Comparison of the t-SNE visualizations of the (**a**) original and (**b**) fisheye-preprocessed datasets for a specific typhoon (*Nock-ten 2016*). The numbers 0 to 23 represent the 24 hourly data points from the appearance to the disappearance of the typhoon. The points are color-coded according to the ground truth.

To further explore the effect of the fisheye preprocessing on the typhoon intensity classification, we next applied t-SNE to the satellite image dataset itself. As shown in Fig. 4, we compared the t-SNE distributions of the original and fisheye-preprocessed images. To simplify the visualization here, the figure shows only the data points and images associated with Typhoon Nock-ten, which occurred in late December 2016. As seen in Fig. 4(a), the temporally continuous data points were very close to each other in the two-dimensional space because, from a bird’s eye view, the shape of the typhoon clouds in the original images hardly changed over 6 hours. Therefore, if these similar images actually belonged to different intensity classes, the CNN would misclassify either of them. On the other hand, as seen in Fig. 4(b), the temporally continuous data points were located farther apart in the fisheye-preprocessed images . In addition, by comparing the images shown in the figure, we can easily see some differences such as the clarity of the eye location, the size, and the shape of the eyewall. Thus, we concluded that the fisheye preprocessing could highlight the uniqueness of the typhoon shape at the moment an image was captured, which consequently enabled the CNN to precisely estimate the typhoon intensity class from the satellite imagery.

**Figure 5:**
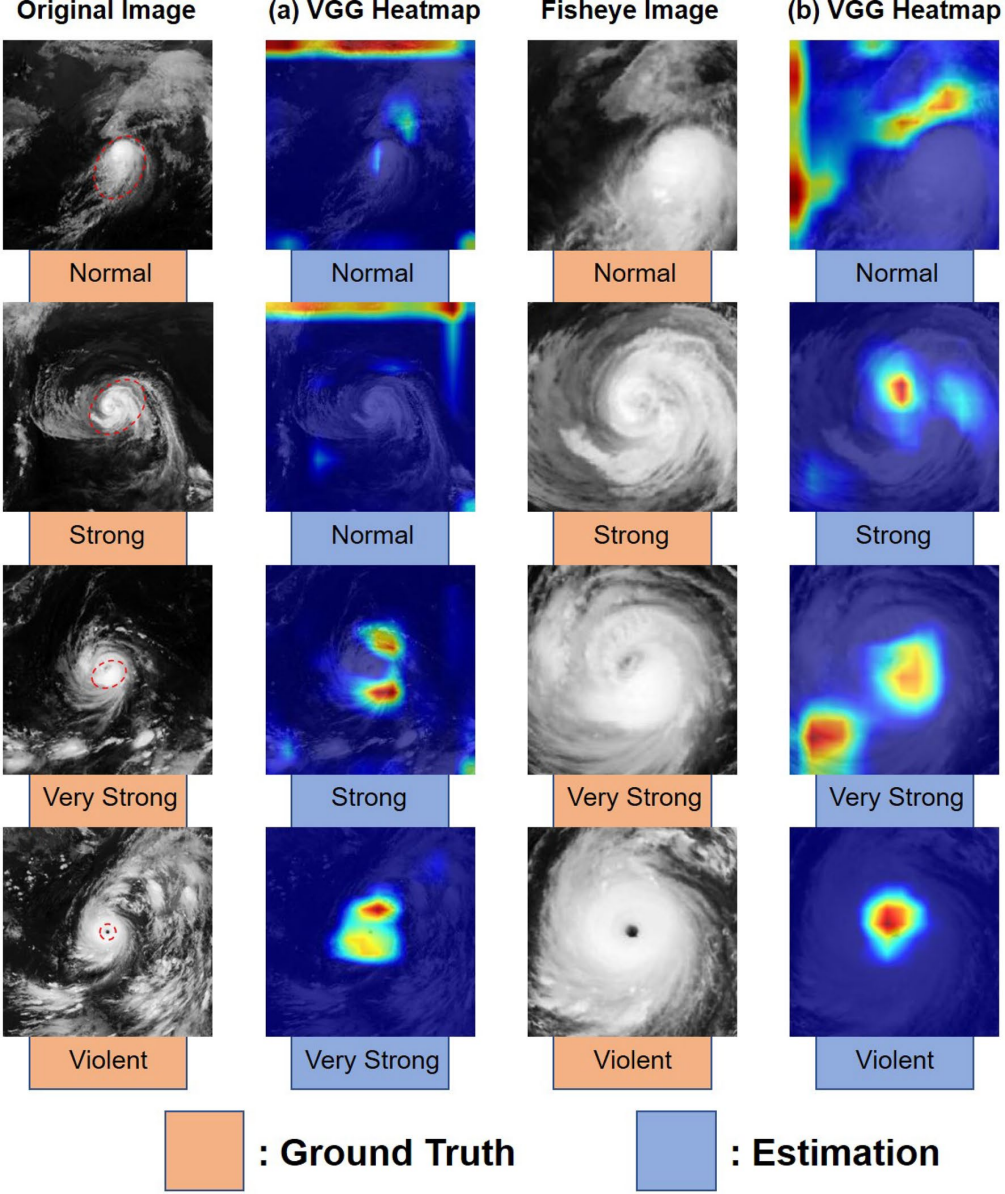
Comparison of the Grad-CAM heatmaps from the final convolutional layers of the two models: VGG-16 with the (**a**) original and (**b**) fisheye-preprocessed datasets. We applied Grad-CAM to each model after training over 25 epochs. The important regions in the images for estimating the typhoon intensity class are shown in red. The areas outlined by the red dashed lines in the original images indicate the unique features of each cloud pattern.

### Grad-CAM visualization

Next, we performed sensitivity analyses of the two models by using Grad-CAM^42^, which highlights the localized regions in a satellite image that are useful for typhoon intensity classification. Figure 5 shows a comparison of the Grad-CAM heatmaps between the two models for the estimation results of the four intensity classes.

Eye patterns are seen at the mature stage, and they often show a clear eye at the typhoon center, which is a distinctive feature of the violent intensity class^5^. As seen in the figure, for violent typhoons, Grad-CAM highlighted the eye in red as the most critical region for intensity classification in the fisheye-preprocessed images; in contrast, the highlighting of the eye was not nearly as strong in the original images. Therefore, our best model, VGG-16 with the fisheye-preprocessed images, could perfectly capture a key feature of violent typhoons.

Another key feature is cloud dense overcast (CDO) patterns, which mainly appear in very strong or violent typhoons and are defined by a dense, solid-looking mass of clouds covering the typhoon center. The Dvorak technique determines the typhoon intensity according to the CDO size, and the shape can be round, oval, angular, or irregular^5^. The CDO often lies within the curve of a typhoon’s curved cloud band. As seen in the images for the very strong typhoon in Fig. 4, the CNN trained with the original images focused on the outer band, far from the image center, and incorrectly estimated the intensity. On the other hand, the CNN trained with the fisheye images focused on the CDO directly below the image center and correctly estimated the intensity. Therefore, our best model captured a key feature of very strong typhoons more accurately than the original model did.

Curved band patterns, which consist of a spiral cloud band wrapped around the typhoon center and have a tiny eye in the case of a strong or very strong typhoon, are commonly observed in satellite images, especially during the development stage of a typhoon. For this kind of pattern, the Dvorak technique determines the typhoon intensity according to the outer band’s length and the amount of dense overcast surrounding the typhoon center^5^. The fisheye heatmap for the strong typhoon image in Fig. 5 shows that Grad-CAM highlighted the spiral band near the typhoon center and another one in the southwest part of the image. In other words, the best model captured a feature of strong typhoons that is used in the Dvorak technique.

Shear patterns, which are associated with the normal typhoon intensity class, appear in the development stage when vertical shear prevents cold clouds from bending around the cloud system center as they do in curved band patterns^5^. These patterns also appear in the decay stage because of increasing vertical shear. In a satellite image, a shear pattern can be recognized as dense overcast appearing off to the side of the typhoon center. As seen in Fig. 5, Grad-CAM partially highlighted the dense overcast on the right side of the fisheye-preprocessed image. Therefore, our best model could capture some shear patterns.

### Limitation of data-driven approach alone/importance of domain-knowledge integration

In this study, we used a CNN to automate the Dvorak technique for typhoon intensity classification from a single satellite image. We found that the network achieved high discrimination performance by using image preprocessing based on domain knowledge of meteorology, with a classification accuracy of 76.8%.

As seen in Supplementary Table S2a, we confirmed that random split validation enabled even VGG with the original satellite images to achieve a high accuracy of 78.7%. This result implies that sequential split validation should be a hard problem. On the other hand, interestingly, Supplementary Table S2b indicates that the test accuracy of VGG with the fisheye-preprocessed images in the random split validation was also 78.7%. By comparing with the results listed in Table 2b for the sequential split validation, we conclude that the fisheye preprocessing may be able to extract almost the maximum amount of rough information about typhoon intensity from satellite images.

As seen in Supplementary Table S3, we also confirmed that even VGG trained with only two recent years of the fisheye dataset achieved a test accuracy of almost 70%. From the results in the tables, we can also see that VGG became less likely to mistake a violent typhoon for a normal or strong typhoon as the amount of data available for training increased.

Note that an approach of using satellite images created by naively enlarging and cropping the center of the original images could not achieve the same accuracy. Supplementary Figure S3 shows the relation between the cropped image size and the classification accuracy of VGG-16. Through numerical validation, we cropped satellite images in ranges from $1000\times 1000$ km to $2600\times 2600$ km (i.e., the original size) around the centers of the original images. The figure reveals that the optimal cropped size was around $1300\times 1300$ km. The cropped images were somewhat similar to the fisheye-preprocessed images, but VGG-16 had lower accuracy for any cropped size. Naive cropping might cut even parts of the image that meteorology experts would consider necessary for the intensity classification. On the other hand, the fisheye preprocessing could retain some of these parts in a compressed state while emphasizing the central parts of the images.

**Figure 6:**
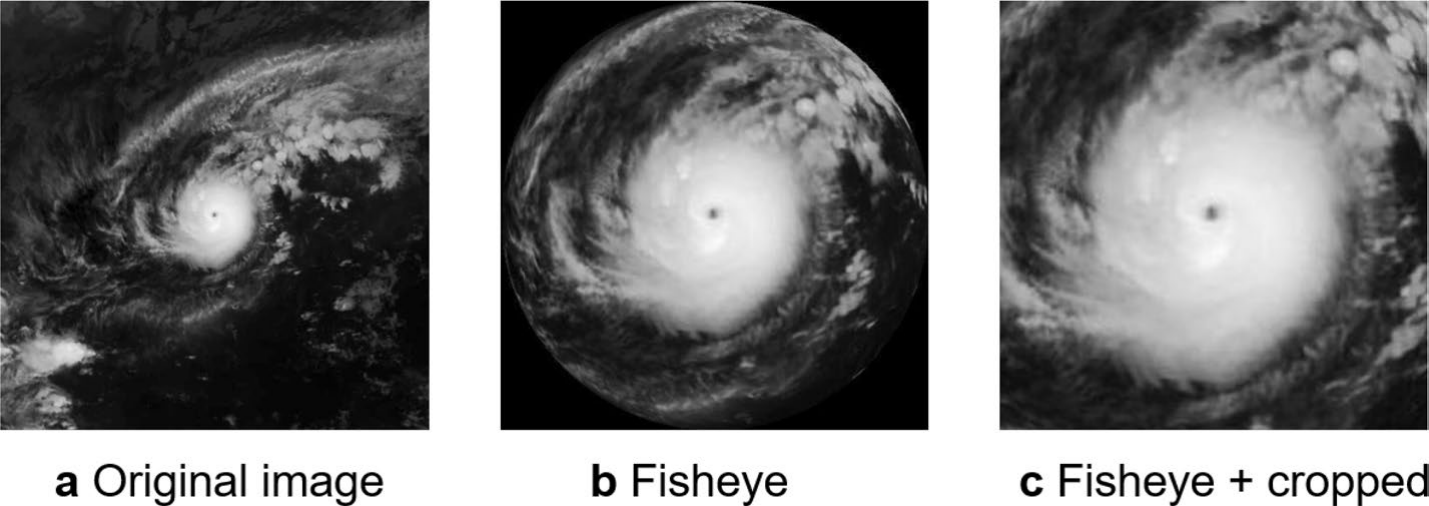
Procedure of fisheye image preprocessing. We first applied fisheye distortion to (**a**) an original image. We then cropped (**b**) the fisheye-distorted image into (**c**) a square one.

Similarly, Supplementary Fig. S4 shows that the fisheye distortion effect alone could not achieve the classification accuracy of 76.8%. This was because the fisheye distortion generated a circular artifact, as shown in Fig. 6b. In preliminary experiments using images like this, VGG-16 identified this artifact as a feature for typhoon intensity classification; hence, the cropping was applied to eliminate the artifact.

The regression results in Fig. 2 and Supplementary Fig. S2 show that the statistical difference between the JMA best-track data and the VGG outputs from the fisheye-preprocessed images was around 11 kt. Olander and Velden^49^ reported that the intensity RMSE between ADT and Joint Typhoon Warning Center (JTWC) best tracks is 11.24 kt in the northwest Pacific. There are also considerable differences between the best-track data of the JTWC and JMA^52^, and we should thus be careful in comparing the proposed model and the existing methods. For example, Ito et al.^50^ reported that the statistical difference between aircraft observations and the JMA best tracks for Typhoon No. 21 in 2017 was 10-15 hPa. Shimada et al.^51^ also reported that the intensity RMSE between radar observations and JMA best tracks for a typhoon approaching Japan was approximately 8 hPa. Hoshino and Nakazawa^52^ reported that the difference between the brightness temperatures of the Tropical Rainfall Measuring Mission (TRMM) Microwave Imager (TMI) and the JMA best track is approximately 10 kt in the northwest Pacific. Summarizing the above, as there is no true value of wind speed, it is difficult to say that our best model outperforms the existing methods such as ADT^32^ or SATCON^33^, but it can be said that it has a comparable level of performance. Moreover, to the best of our knowledge, there is no other simple, powerful method that can estimate typhoon intensity from only a single satellite image by using a CNN as we do. To further reduce the RMSE, the categories of the classification model might be more precisely set to 5-kt intervals.

Generally, data-driven approaches using CNNs select significant features embedded in images in a bottom-up procedure. In other words, without any preprocessing based on domain knowledge, a CNN would evaluate each pixel of an original image equally for classification. As shown in Fig. 5, the Grad-CAM results suggested that simple training with the original satellite images could not give the CNN the same discriminatory capability as meteorological experts. For example, the eye, a characteristic feature of a typhoon, was represented by at most one pixel in the original satellite images. As a result, the CNN lost this meteorologically important feature through repeated convolution and pooling, because the proportion of the eye in the original images was too small. On the other hand, the fisheye distortion, which weighted the center of the satellite image, allocated roughly $10 \times 10$ pixels to represent the eye. Thus, the fisheye-preprocessed images allowed the CNN to recognize the eye as a crucial region for intensity classification. Supplementary Fig. S5 shows how the combination of the cropping and fisheye preprocessing improved the classification accuracy. This top-down approach based on domain knowledge could indirectly instruct the CNN on which parts of a typhoon image to focus on meteorologically.

Here, we acknowledge a limitation of our study: that only the satellite images were used as inputs to the CNN to automate the Dvorak technique. We found that some typhoons had different intensity classes even though their satellite images were still nearly indistinguishable from each other after the fisheye preprocessing was applied to them. The use of other weather data, such as a typhoon’s current location, the sea surface temperature, and the number of days elapsed since a typhoon’s appearance could further improve the classification accuracy.

Moreover, in the same manner as in a previous study^18^, we didn’t use any temporal information about the intensity classes. For example, we could implement a more accurate classifier by incorporating the time-series properties of a typhoon through information about its intensity six hours previously. Specifically, we can use the difference image obtained by subtracting a satellite image recorded six hours previously from a current one as an additional input to the CNN. If the CNN can successfully extract changes in cloud dynamics from the difference image, then we believe that this model can work even in the operational phase when the best-track intensity is not available. In a future work, we should thus design a deep learning architecture to classify the typhoon intensity by using not only satellite images but also observation data like that mentioned above.

## Dataset

### Typhoon images

In this study, we used infrared satellite images of typhoons observed in the northwest Pacific Ocean by a geostationary meteorological satellite, Himawari^43^. The Himawari observation data used in this study was around 10.4 $\upmu$m (IR1). The images were obtained from a digital typhoon data repository^44^. All the original images have a side length of 2600 km, with the center of the typhoon described in a best-track manner, and the image size is $512\times 512$ pixels. The typhoon images from 2005 to 2014 were used for training the CNN, while those from 2015 to 2016 were used for testing. Table 1 summarizes the JMA definitions of the four typhoon intensity classes^45^, which are the classification target for the CNN output. Following these definitions, we converted the best-track maximum sustained wind (10-min mean) associated with a typhoon, which was obtained from the Regional Specialized Meteorological Center (RSMC) Tokyo^46^, into the intensity. Subsequently, the typhoon images were labeled with the corresponding intensity classes.

### Image preprocessing

Fisheye distortion allows us to enhance an area of interest in greater detail while preserving the surrounding environment. To emphasize the center of a typhoon, we applied fisheye distortion to the original satellite images, like the one shown in Fig. 6a, by using the following equations:

2 $$\begin{aligned} \text {dst}({x,y})= & {} \text {src} \left( \frac{Ax+ 0.5\text {w}}{s},\frac{Ay+ 0.5\text {h}}{s}\right) , \end{aligned}$$

3 $$\begin{aligned} {A}= & {} \frac{\left( 1-\frac{2}{\pi }\arccos {\frac{\sqrt{x^2+y^2}}{r}} \right) r}{\sqrt{x^2+y^2}}, \end{aligned}$$

4 $$\begin{aligned} r= & {} \min {(0.5w,0.5h)}. \end{aligned}$$

Here, the width *w* and height *h* were both 512, and the radius *r* was defined to be half the width or height, whichever was smaller. The variable *s* represents the expansion rate, for which the optimal parameter was determined by maximizing the average accuracy of the model given the fisheye dataset, as shown in Supplementary Fig. S5. By applying the fisheye distortion to Fig. 6a, we obtained circular typhoon images like the one shown in Fig. 6b. Next, we cropped a $360\times 360$ pixel area from the center, as shown in Fig. 6c. When using the second model shown in Fig.1b, we applied above processing which consist of the fisheye and trim processing to all images. Note that we used images that were normalized and resized to $224\times 224$ pixels for both models.

### VGG-16

We used a VGG-16 model that had previously been trained on the ImageNet dataset^47^. We only trained the FC layers of the VGG-16 model, except for the convolutional layers. The first FC hidden layer consisted of 1024 nodes, while the second one consisted of 512 nodes. In training the model, we used a 30% dropout rate for the first and second FC hidden layers, with a ReLU activation function. The model was trained with the stochastic adaptive moment (Adam) optimizer^48^ with a learning rate of 1e-3 (0.001) and a total of 50 epochs.

## Methods

### Performance evaluation

The accuracy, precision, recall (also known as sensitivity), and F1 score were used to assess the performance of each model on the test dataset. From a disaster-prevention perspective, we emphasized the sensitivity to typhoons, which are the most severe storms and can devastate people’s lives.

### t-SNE

To visualize the data associated with the typhoon satellite imagery in a two-dimensional space, we used a nonlinear dimension reduction method called t-distributed stochastic neighbor embedding (t-SNE)^41^. First, we applied t-SNE to the feature maps of the last convolutional layer in each of the two models, given the test data. Second, we applied t-SNE to both the original satellite images and the fisheye-preprocessed images of Typhoon *Nock-ten 2016*.

### Grad-CAM

Grad-CAM^42^ visualizes the important regions of an input image via a heatmap by using the gradient information of the last convolutional layer. The pooling layer outputs feature maps from an input image, and the FC layer converts the feature maps to probability scores for each class. The gradient is a coefficient that indicates the magnitude of the change in the probability score when part of the image is changed. The gradient is thus large for parts of the image that strongly influence the class determination. We applied Grad-CAM to the outputs of the final convolution layer of each model and obtained a heatmap of each typhoon image.

## Supplementary information

Supplementary material 1 (pdf 970 KB)

## Data Availability

The datasets and code used in this study are respectively available from the seventh and first authors on request.
