# Supplementary material for: Domain knowledge integration into deep learning for typhoon intensity classification
Source: Sci Rep. 2021 Jun 21;11:12972. doi: 10.1038/s41598-021-92286-w (PMC8217498; doi:10.1038/s41598-021-92286-w)
Supplement: Supplementary file 1 — Supplementary material 1 (pdf 970 KB) [file 41598_2021_92286_MOESM1_ESM.pdf]

Supplementary information

Title: Domain Knowledge Integration into Deep Learning for Typhoon Intensity Classification

Authors: Maiki Higa, Shinya Tanahara, Yoshitaka Adachi, Natsumi Ishiki, Shin Nakama, Hiroyuki Yamada, Kosuke Ito, Asanobu Kitamoto, and Ryota Miyata

Supplementary Table S1. Detailed hyperparameters of the proposed models.

Hyperparameter	Value
Batch Size	16
Peak Learning Rate	1E-03
Learning Rate Decay	Linear
Max Steps	7175
Adam β_1	0.9
Adam β_2	0.999

Supplementary Table S2. Confusion matrices and classification results of random split validations using VGG-16 with the (a) original and (b) fisheye-preprocessed datasets. In this cross-validation, the number of each intensity class in the test dataset was set to be approximately the same as in the 2015-2016 dataset.

(a) Original dataset + VGG-16 (Accuracy: 78.7%)

Actual Category \ Prediction	N	S	VS	V	Precision	Recall	F1-score
**Normal (N)**	683	58	3	0	0.87	0.92	0.89
**Strong (S)**	89	153	43	0	0.63	0.54	0.58
**Very Strong (VS)**	13	33	107	16	0.66	0.63	0.65
**Violent (V)**	0	0	9	28	0.64	0.76	0.69

(b) Fisheye-preprocessed dataset + VGG-16 (Accuracy: 78.7%)

Actual Category \ Prediction	N	S	VS	V	Precision	Recall	F1-score
**Normal (N)**	643	70	0	0	0.87	0.90	0.89
**Strong (S)**	82	195	22	0	0.60	0.65	0.63
**Very Strong (VS)**	11	58	107	7	0.76	0.58	0.66
**Violent (V)**	0	2	11	27	0.79	0.68	0.73

Supplementary Table S3. Confusion matrices and classification results for VGG trained with several-year typhoon data selected from 2005-2014 in reverse chronological order. The input was the fisheye-preprocessed images shown in Fig. 6 (c). In the same way as Results in our main article, 2015-2016 data were used for testing.

(a) 2013-2014 for training (Accuracy: 69.6%)

Actual Category \ Prediction	N	S	VS	V	Precision	Recall	F1-score
**Normal (N)**	622	37	0	0	0.74	0.94	0.83
**Strong (S)**	170	111	22	0	0.49	0.37	0.42
**Very Strong (VS)**	43	72	100	12	0.74	0.44	0.55
**Violent (V)**	1	5	14	26	0.68	0.57	0.62

(b) 2011-2014 for training (Accuracy: 69.9%)

Actual Category \ Prediction	N	S	VS	V	Precision	Recall	F1-score
**Normal (N)**	605	51	3	0	0.78	0.92	0.84
**Strong (S)**	141	123	39	0	0.49	0.41	0.44
**Very Strong (VS)**	30	73	108	16	0.65	0.48	0.55
**Violent (V)**	0	4	15	27	0.63	0.59	0.61

(c) 2009-2014 for training (Accuracy: 71%)

Actual Category \ Prediction	N	S	VS	V	Precision	Recall	F1-score
**Normal (N)**	597	59	3	0	0.81	0.91	0.85
**Strong (S)**	123	141	39	0	0.49	0.47	0.48
**Very Strong (VS)**	21	83	116	7	0.65	0.51	0.57
**Violent (V)**	0	3	20	23	0.77	0.50	0.61

(d) 2007-2014 for training (Accuracy: 72.2%)

Actual Category \ Prediction	N	S	VS	V	Precision	Recall	F1-score
**Normal (N)**	617	41	2	0	0.79	0.93	0.86
**Strong (S)**	141	109	53	0	0.53	0.36	0.43
**Very Strong (VS)**	22	55	142	8	0.66	0.63	0.64
**Violent (V)**	0	2	19	25	0.76	0.54	0.63

(a) Original dataset + LeNet

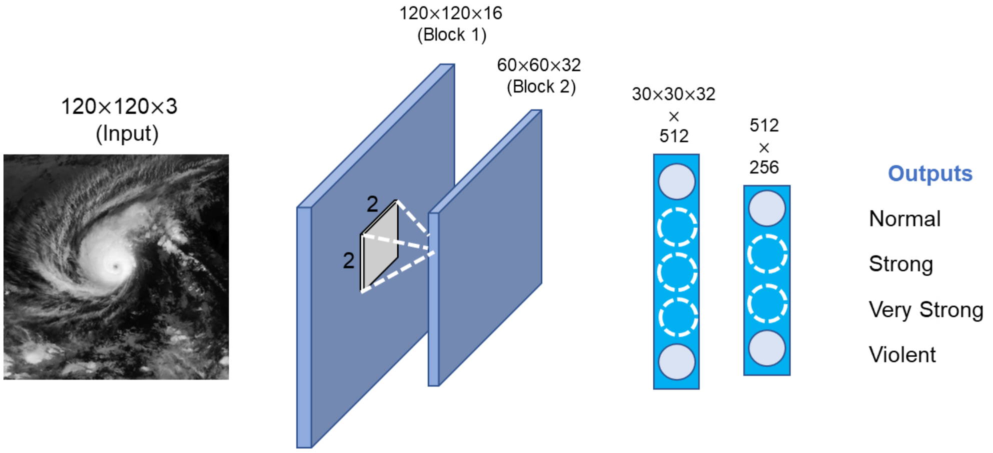

(b) Original dataset + LeNet (accuracy: 55.7%)

Actual Category \ Prediction	N	S	VS	V	Precision	Recall	F1-score
**Normal (N)**	519	112	28	3	0.67	0.78	0.72
**Strong (S)**	150	100	47	6	0.34	0.33	0.34
**Very Strong (VS)**	87	65	67	8	0.42	0.30	0.35
**Violent (V)**	14	13	16	3	0.15	0.07	0.09

Supplementary Figure S1. (a) LeNet architecture used in the preliminary experiment for typhoon intensity classification from satellite images. For comparison, we adopted the same manner of sequential-split validation as Results in our main article (i.e., 2005-2014 for training, and 2015-2016 for testing). **(b)** Confusion matrix and classification results for LeNet with the original dataset.

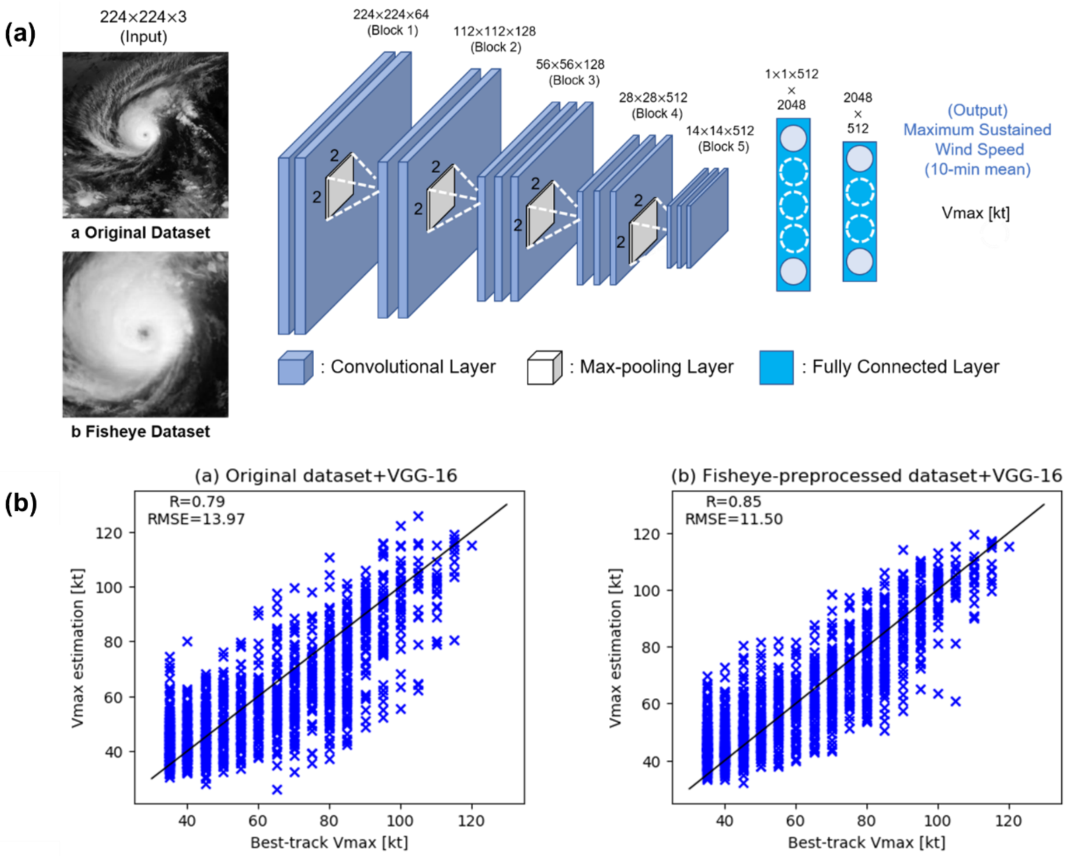

(c)

Prediction / Actual Category	N	S	VS	V	Precision	Recall	F1-score
**Original dataset + VGG-16 (Accuracy: 67.7%)**							
Normal (N)	610	47	2	0	0.75	0.93	0.83
Strong (S)	164	118	20	1	0.46	0.39	0.42
Very Strong (VS)	40	84	90	13	0.68	0.40	0.50
Violent (V)	1	7	20	18	0.56	0.39	0.46

Prediction / Actual Category	N	S	VS	V	Precision	Recall	F1-score
**Fisheye-preprocessed dataset + VGG-16 (Accuracy: 72.1%)**							
Normal (N)	596	63	0	0	0.83	0.90	0.87
Strong (S)	111	153	39	0	0.51	0.50	0.51
Very Strong (VS)	10	81	118	18	0.66	0.52	0.58
Violent (V)	1	1	21	23	0.56	0.50	0.53

Supplementary Figure S2. (a) Architecture of VGG-16 regression to estimate the speed of maximum sustained wind (MSW, 10-min mean) associated with a typhoon directly from satellite images. We adopted the same manner of sequential split validation as in the typhoon intensity classification of our main article (i.e., data from 2005 to 2014 for training and those from 2015 to 2016 for testing). **(b)** Scatter plots of VGG-16 regressions to estimate the MSW values from the (left-side) original and (right-side) fisheye-preprocessed satellite images, respectively. **(c)** Confusion matrices of the typhoon intensity classifications based on the VGG-16 regressions. The wind speeds estimated by each VGG regression were converted into 4 categories according to the typhoon intensity definitions in Table 1.

(a) VGG-16 with cropped images

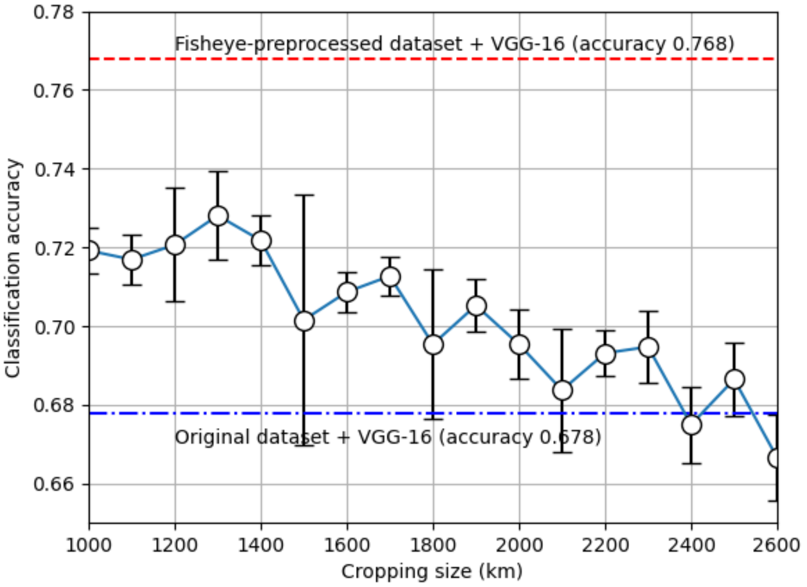

(b)

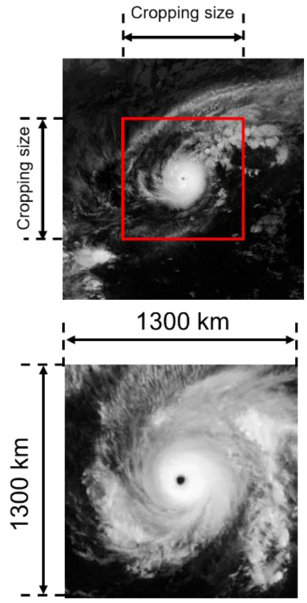

(c) VGG-16 with 1,300-km cropped images (accuracy: 74%)

Prediction / Actual Category	N	S	VS	V	Precision	Recall	F1-score
**Normal (N)**	600	57	2	0	0.81	0.91	0.86
**Strong (S)**	120	151	32	0	0.55	0.50	0.52
**Very Strong (VS)**	16	64	143	4	0.72	0.63	0.67
**Violent (V)**	1	2	23	20	0.83	0.43	0.57

Supplementary Figure S3. (a) Relationship between the classification accuracy (average of five runs with standard deviation) and the cropped size for VGG-16 with cropped satellite images. In this numerical validation, the side length of the cropped square was varied in intervals of 100 km from 1,000 km to 2,600 km (original size) around the center of the image. **(b)** Square cropping **(upper)** and an example of a 1,300-km cropped image **(lower)**, which achieved the best accuracy of 0.74, as shown in **(a)**. **(c)** Confusion matrix and classification results for VGG with the 1,300-km cropped images.

(a) VGG with the fisheye-distorted images

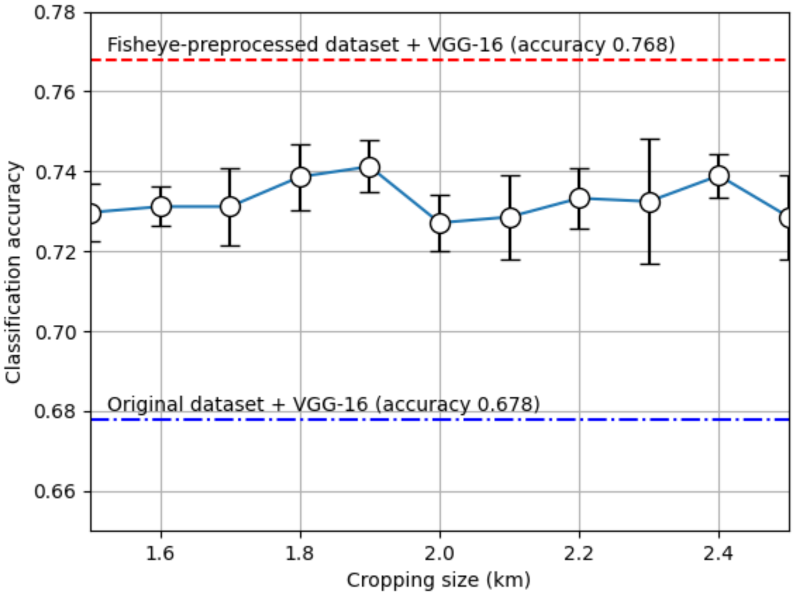

(b)

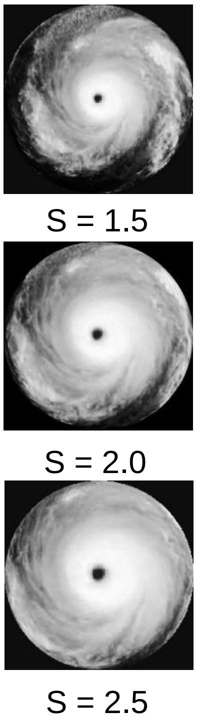

(c) VGG with the fisheye-distorted images with S = 1.9 (accuracy:75.4)

Prediction / Actual Category	N	S	VS	V	Precision	Recall	F1-score
**Normal (N)**	595	63	1	0	0.86	0.90	0.88
**Strong (S)**	81	180	42	0	0.56	0.59	0.58
**Very Strong (VS)**	14	73	130	10	0.68	0.57	0.62
**Violent (V)**	0	3	17	26	0.72	0.57	0.63

Supplementary Figure S4. (a) Relationship between the classification accuracy (average of five runs with standard deviation) and the fisheye distortion parameter *s*, i.e., the central expansion rate. The vertical axis indicates the accuracy, and the horizontal axis indicates the expansion rate, from 1.5 to 2.5 in intervals of 0.1. **(b)** Training samples of typhoon images that were subjected to preprocessing. **(c)** Confusion matrix and classification results for the CNN model that achieved the highest accuracy, with a fisheye

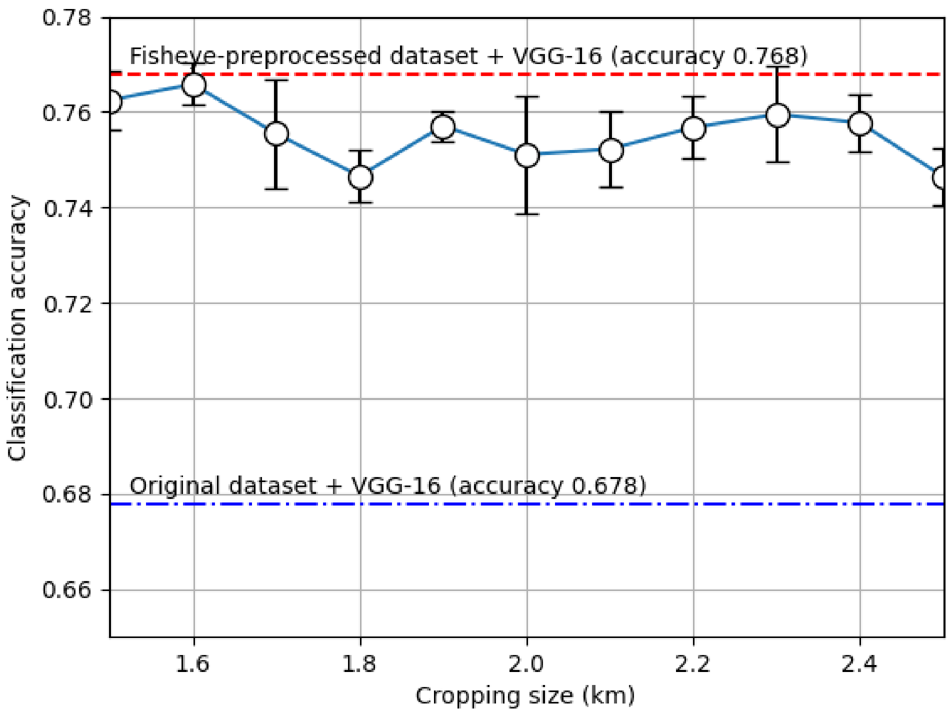

Supplementary Figure S5. Relationship between the classification accuracy (average of five runs with standard deviation) and a combination of the fisheye distortion and cropping parameters. The vertical axis indicates the accuracy of the test classification by CNNs trained on the cropped and fisheye-preprocessed images. The horizontal axis indicates the expansion rate described for the first preprocessing step shown in Fig. 6.
